# Palindromic Rheumatism: Just a Pre-rheumatoid Stage or Something Else?

**DOI:** 10.3389/fmed.2021.657983

**Published:** 2021-03-25

**Authors:** Raimon Sanmartí, Beatriz Frade-Sosa, Rosa Morlà, Raul Castellanos-Moreira, Sonia Cabrera-Villalba, Julio Ramirez, Georgina Salvador, Isabel Haro, Juan D. Cañete

**Affiliations:** ^1^Arthritis Unit, Rheumatology Service, Institut d'Investigacions Biomèdiques August Pi i Sunyer (IDIBAPS), Hospital Clinic de Barcelona, Barcelona, Spain; ^2^Hospital Central del Instituto de Previsión Social, Asunción, Paraguay; ^3^Hospital de Clínicas Universidad Nacional de Asunción, Asunción, Paraguay; ^4^Rheumatology Department, University Hospital Mutua Tarrasa, Barcelona, Spain; ^5^Unit of Synthesis and Biomedical Applications of Peptides, Institute of Advanced Chemistry of Catalonia, Consejo Superior de Investigaciones Científicas (IQAC-CSIC), Barcelona, Spain

**Keywords:** palindromic rheumatism, rheumatoid arthritis, ACPA, pre-RA, management

## Abstract

Palindromic rheumatism (PR), a unique clinical entity, has a characteristic clinical presentation with a relapsing/remitting course. It is established that most patients with PR evolve to chronic disease, of which rheumatoid arthritis (RA) is by far the most common. The relationship between PR and RA is unclear, with similarities and differences between the two, and not all patients evolve to RA in the long-term. Therefore, PR is clearly a pre-RA stage for most, but not all, patients. Autoimmunity plays a substantial role in PR, with the same characteristic autoantibody profile observed in RA, although with some differences in the immune response repertoire. Autoinflammation may also be relevant in some cases of PR. Prognostic factors for RA progression are identified but their exact predictive value is not clear. There are several unmet needs in PR, such as the diagnostic criteria and clinical case definition, the pathogenic mechanisms involved in the unusual clinical course, and the evolution to RA, and our understanding of the therapeutic strategy that could best avoid progression to persistent and potentially destructive arthritis.

## Introduction

A-36-year-old female office worker presented in the Arthritis Unit with an 8-month history of intermittent joint pain and swelling. The family physician had prescribed naproxen during flares without significant improvement. The first blood test disclosed a rheumatoid factor (RF) of 40UI (NV <20) and a negative antinuclear (ANA) test, without other significant changes and with normal levels of CRP, ESR and uricemia. No familial occurrence, arthritis or other immune-mediated disease was found and no comorbidities were recorded. A detailed clinical history of the joint symptoms revealed that the patient had joint attacks that initiated abruptly (maximum pain in 2 h) and disappeared after 24–36 h. Between attacks, the patient was symptom free. The interval between flares was irregular but the patient reported 2–3 episodes per month, some of which were disabling. The most frequent joints affected were the wrists and metacarpophalangeal joints, but also the shoulders and ankle on a few occasions. Swelling was observed in several but not all attacks, especially in the hand joints, accompanied by erythema. No fever was documented. All but one of the attacks were monoarticular; the other affected two joints simultaneously. Laboratory data in our hospital confirmed RF positivity and anti–citrullinated protein antibody (ACPA) (CCP2 test) positivity at high titers (456 UI). Treatment with hydroxychloroquine was begun.

The most probable diagnosis of this patient is palindromic rheumatism, due to the typical presentation, but what other diagnoses should be considered? Can the evolution or prognosis be ascertained? Can the evolution to persistent arthritis, such as rheumatoid arthritis (RA) or other rheumatic diseases be predicted? What are the best management and therapeutic strategies?

In this article, we try to analyze these questions in accordance with scientific evidence and our own experience, focusing on the enigmatic relationship between PR and RA.

## Brief Historical Perspective

The first patient was recognized in 1928 by Philip S Hench in the Mayo Clinic, Rochester, USA. A 21-year-old women presented with hundreds of attacks of pain and swelling in different joints of very short duration (between 12 and 36 h), in general involving one joint at a time, with the patient being symptom-free between attacks. Hench observed 34 similar cases and, together with Edward Rosenberg, reported them in 1944 (1). The authors stated the attacks were unpredictable and, in some cases, periarticular/para-articular inflammation was observed. The clinical course varied considerably but no residual joint damage was observed. The authors analyzed entities with a similar clinical presentation and concluded that this was a unique, undescribed entity. Since no etiologic factor was found, and terms such as intermittent, recurrent or remitting rheumatism seemed to be unsatisfactory because they were too unspecific, the authors proposed “palindromic rheumatism” as a name. Palindromic is derived from the Greek *palin dromein* and means “to run back” or simply returning or recurring. The term was used by Hippocrates to denote erysipelas and other conditions that tend to repeat in the same individual. One of the article's advisors said, “*What was good enough for Hippocrates ought to be good enough for you*.” Since this first description, the designation of PR has remained until the present.

Hench and Rosenberg separated PR from RA due to the differentiating clinical relapsing/remitting course. Surprisingly, none of the patients in the original series evolved to persistent, chronic arthritis even after a long-term follow-up. By contrast, all subsequent series found that a significant proportion of patients evolved to RA or other chronic rheumatic disease. In 1959, Ansell and Bywaters, in a clinical meeting of the Heberden Society, presented a study in 28 patients with PR that showed that 18 patients evolved to chronic polyarthritis (13 were RF positive), and suggested that PR was merely a form of pre-RA, since almost all patients would progress to RA in the long term (2). However, the same authors also found that one patient remained as PR after 24 years of follow up.

The discovery that most, but not all, patients evolved to RA in the long-term (2, 3) and that a significant proportion had the same autoantibody profile: RF (4) and, more recently, ACPA (5), reinforced the close relationship between PR and RA. However, this relationship is not clear, and it uncertain whether PR is simply part of the RA continuum, or represents a pre-rheumatoid stage or is a separate entity (6–8). From today's perspective, PR probably represents a syndrome rather than a disease as cases with a similar phenotype of palindromic-like arthritis may display different mechanistic pathways with a different evolution and prognosis, although a high proportion of patients evolve to RA.

## Diagnosis of Palindromic Rheumatism

The diagnosis of PR is clinical, with a presentation of very short-lasting joint attacks with a relapsing/remitting course and no other explanation of the clinical symptoms. However, as there are no validated classification or diagnostic criteria in PR, there are wide variations in the clinical phenotype described in different series, although in all series a characteristic clinical presentation is described: patients are symptom free between attacks, joint swelling frequently presents with periarticular inflammation, and the recurrent episodes last <3 days in most cases (between 12–48 h in the majority) (6). The differential diagnosis of PR is quite wide due to the diseases that can cause an intermittent pattern of arthritis (9). In our opinion, the very spontaneous short-lasting episodes are a hallmark of PR and may differentiate it from other arthritis with an intermittent course (10) (Table 1). Whipple disease and autoinflammatory disorders such as familial Mediterranean fever (FMF) may have a similar clinical presentation of very short-lasting attacks (11, 12). The attacks only occasionally last for more than a week, a finding that prompted Barbero and Pasieri to determine this as the maximum permissible to consider in the diagnosis of PR (13). The distribution of the joint involved is similar to that seen in RA, with some differences between studies (6, 14–17) (Table 2).

**Table 1:** Differential diagnosis of intermittent arthritis.

**Disease**	**Joint pattern**	**Duration**
Palindromic rheumatism	Monoarticular Hand predominance	Hours-days
Gout	Monoarticular Feet, LL	Days
Calcium pyrophosphate deposition disease (pseudogout)	Mono-oligoarticular Knee, wrist	Days-week
Reactive arthritis	Mono-oligoarticular Asymmetric Lower limb dominance	Weeks-month
Arthritis associated with inflammatory bowel disease.	Mono-oligoarticular Asymmetric Large joints (LL) Axial manifestation	Days-months
Whipple's disease	Mono-oligoarticular Large joints (LL) Axial manifestation possible	Days
Behçet disease	Mono-oligoarticular Asymmetric Large joints	Days-weeks
Sarcoidosis	Oligo-polyarticular Symmetric	Weeks
Familial Mediterranean Fever	Monoarticular Large joints(LL)	Hours-days
TRAPS	Arthralgia of large joints	Days-weeks
HIDS	Large joints (LL)	Days
Celiac disease	Oligo-polyarthritis Asymmetric LL dominance Axial manifestation possible	Weeks
Intermittent hydrarthrosis	Mono-oligoarticular (knee)	Days
Relapsing polychondritis	Oligo-polyarticular asymmetric	Days-weeks
Hyperlipidemia type II-IV	Oligoarticular Small and large joints	Days-weeks
Hereditary angioedema	Periarticular with oedema	Days
Lyme's arthritis	Mono-oligoarticular	Weeks-months
Allergic eosinophilic synovitis	Oligo-polyarticular	Weeks

LL, Lower limbs; TRAPS, tumor necrosis factor receptor-associated periodic syndrome; HIDS, Hyper immunoglobulin-D syndrome.

*Modified from Cabrera-Villalba and Sanmarti (10)*.

**Table 2:** Distribution of the joints involved in PR in different series.

	**Guerne et al. (6)**	**Gonzalez-Lopez et al. (15)**	**Powell et al. (14)**	**Khabazzi et al. (17)**	**Cabrera-Villalba et al. (16)**
Year (reference)	<1987*	2000	2008	2012	2014
Number of patients	227	113	48	69	54
MCP (%)	91	54	81	51	83
PIP (%)		24		41	85
Wrist (%)	78	64	29	44	85
Knee (%)	64	59	50	68.	78
Shoulder (%)	65	34	27	28	82
Hip (%)	17	9	15	4	NR
Elbow (%)	38	18	17	19	NR
Ankle (%)	50	25	29	22	NR
Feet/MTP (%)	43	18	38	1	NR

*MCP, metacarpophalangeal; PIP, proximal interphalangeal; MTP, metatarsophalangeal*.

Laboratory data show no evidence of biological parameters of inflammation such as CRP or ESR. Some authors suggest that these parameters are frequently increased during the flare, although evidence is scarce and not well-studied (6).

Imaging findings corroborate the absence of articular damage on conventional radiographs. Ultrasound studies between attacks have demonstrated a lack of significant subclinical synovitis in almost all patients (16, 18), confirming the intermittent, not persistent, nature of PR. Interestingly, an extracapsular, periarticular inflammation pattern was observed during clinical attacks, often without accompanying synovitis, which differs from that observed in RA, where intrasynovial inflammation is encountered (18). Other studies mainly found joint synovitis during flares (16, 19). However, the presence of this intrasynovial inflammation in PR during flares has been associated with the presence of ACPA (20) and higher risk for RA progression (18).

The four proposed classification criteria for PR are summarized in Table 3 (6, 13, 15, 21). All suggest that no other diagnosis explains the clinical symptoms and the observation of a clinical episode by a physician in a clinical examination is mandatory. This second statement is of interest, although it may be substituted, in some instances, by a good current mobile imaging photograph. No set of criteria has been validated in clinical studies.

**Table 3:** Proposed diagnostic criteria for palindromic rheumatism.

**Pasero and Barbieri (13)**	**Hannonen et al. (21)**	**Guerne and Weisman (6)**	**Gonzalez-Lopez et al. (15)**
1. A history of brief sudden-onset, recurrent attacks of monoarthritis.	1. Recurrent attacks of sudden-onset mono or polyarthritis of para-articular soft-tissue inflammation lasting from a few hours to 1 week.	1.6-month history of brief-sudden-onset and recurrent episodes of monoarthritis or, rarely, polyarthritis, or of soft tissue inflammation.	1. diagnosis of palindromic rheumatism by a rheumatologist and history of brief sudden onset recurrent episodes of monoarthritis or oligoarthritis and at least two of the following.
2. Direct observation of one attack by a physician.	2. Verification of a least one attack by a physician.	2. Direct observation of one attack by a physician.	2. Direct observation of an attack by the physician
3. More than 5 attacks in the last 2 years.	3. Subsequent attacks in at least three different joints.	3. Three or more joints involved in different attacks.	3. More than 5 attacks in 2 years
4. Three or more joints involved in different attacks.	4. Exclusion of other forms of arthridites.	4. Absence of erosions on radiographs.	4.·3 or more joints involved in the different attacks
5. Negative-X rays, acute phase reactants and rheumatoid factor.		5. Exclusion of other arthritides	5. Normal radiographs
6. Exclusion of other recurrent monoarthritides: gout, chondrocalcinosis, intermittent hydrarthrosis, periodic diseases.			6. Reasonable exclusion of other recurrent monoarthritides

## Pathogenesis of Palindromic Rheumatism: Autoimmunity, Autoinflammation, or Both

It is established that a significant number of patients with PR show a similar serum autoantibody profile to that of RA. RF and ACPA are present in 39–68% of patients in different series, frequently at high titers (5, 16–18, 22–24). Only two Asian studies reported a surprisingly-low prevalence of the two antibodies, suggesting different inclusion criteria or ethnic differences (20, 25). Recently, anti-carbamylated protein (anti-CarP) antibodies have been documented in a quarter of longstanding PR patients (26). An analysis by our group of the antibody immune response to posttranslational modified antibodies (citrullinated or homocitrullinated) showed a more restricted response in PR than in established RA, with less use of the IgM or IgG isotypes (26, 27). This antibody profile resembles that observed in the preclinical phases of RA (28) or in unaffected relatives of patients with RA (29), suggesting the differing B cell immune response, at least in some patients with PR, may play a pathogenetic role, with less probability of evolving to persistent polyarthritis. Whether this antibody profile changes over the PR disease course, with more antigenic expansion or greater isotype distribution, as occurs in RA, in patients who evolve to RA is unclear.

A possible role of autoinflammation in PR has been suggested in view of the clinical presentation of diseases with this relapsing/remitting course that resemble those observed in autoinflammatory disease, such as FMF. MEFV mutations have been observed in 12% of PR patients in a multicenter study, an unexpectedly high prevalence that is more evident in patients without ACPA (23%) (30). This study showed that some cases with a clinical presentation compatible with PR without autoantibodies may be related to autoinflammatory genes, even without other diagnostic criteria.

## Clinical Course and Prognosis

**Figure 1:**
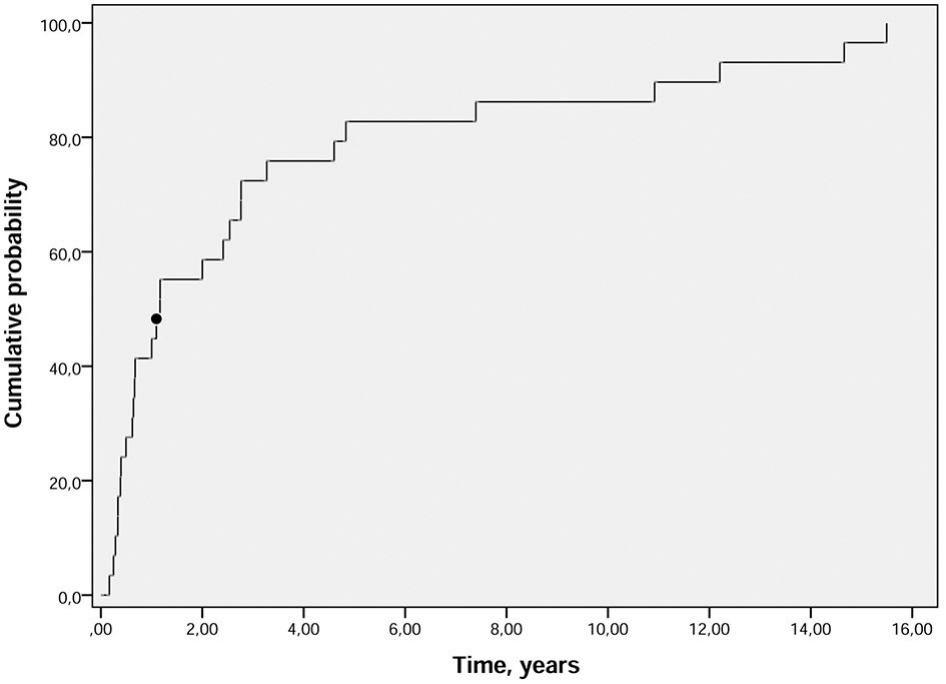
Cumulative probability plot of time (years) from PR diagnosis to RA onset. The median time between PR onset and RA onset was 1.2 (black dot) years (p25–p75: 0.5–3.9). PR, palindromic rheumatism; RA, rheumatoid arthritis (34).

Observational and retrospective series have confirmed the high rate of disease evolution to other chronic rheumatic diseases in PR, with RA by far the most common. The rate of progression to RA is between 10 and 66%, depending on the inclusion criteria, follow-up time and possible therapy (5, 22–24, 31–33). The highest percentage was found in a Scandinavian study of patients followed for more than 20 years (33), although the highest ratio was observed during the first years of evolution (31, 33). It is suggested that 18% of patients with established RA had a history of PR (34). In these patients, RA developed a mean of 1.2 years after initial palindromic symptoms (Figure 1). No particular phenotype emerged in these patients, although palindromic attacks may persist during the disease course of established RA (34). Lower levels of other diseases have been described in the follow-up of patients with PR, including Sjogren syndrome, psoriatic arthritis, vasculitis, myositis, Behçet disease and, most commonly, systemic lupus erythematosus (SLE) (9).

Several prognostic factors for RA progression have been reported, including demographics (female sex) (32), genetics (homozygosity for the shared epitope) (35), the clinical phenotype (hand involvement) (23, 24, 32) and autoantibodies (ACPA and RF) (22–24). ACPA, the most specific serological marker for RA, has been associated with the highest risk for progression to RA, as expected, although we have shown that a significant proportion of ACPA-positive PR patients do not evolve to RA after a long term follow-up (31). Almost all studies were designed to find associations rather than predictive factors, and the limitations included different geographic populations, a small number of cases, a short follow-up time, and different therapies, while some were carried out decades ago. All these factors limit the interpretation of the results and make it difficult to establish the risk for individual progression to RA.

## Management and Therapy

A significant number of antirheumatic drugs have been used in PR, all in case series or observational studies, but no randomized clinical trials have been carried out (36). Therefore, the management of PR is empirical. NSAIDs are used to treat acute attacks with variable results (36). Glucocorticoids may be useful but have been shown to be efficacious only in anecdotal reports (37). Gold salts were effective in reducing the number and intensity of attacks in older studies (3). The same is observed with antimalarials, probably the most widely investigated and used drugs to treat PR to date (36). The efficacy on clinical symptoms is demonstrated in observational studies (38), and currently hydroxychloroquine is probably the drug of choice for PR. One study found that antimalarials may increase the time of progression to RA but not avoid it (15). Methotrexate, the most widely-used drug in the treatment of RA, has not been tested in PR, although a recent observational, uncontrolled study used methotrexate as part of a treat-to-target strategy that resulted in substantial clinical benefits, with low rates of progression to RA (39). A surprisingly high rate of clinical response was observed with rituximab in an Indian survey (40). Colchicine is effective in seronegative PR patients with MEFV mutations (30). A multicenter randomized clinical trial comparing the efficacy of abatacept vs. hydroxychloroquine in recent-onset seropositive PR is ongoing (ClinicalTrials.gov Id: NCT03669367).

## Unmet Needs in Palindromic Rheumatism and Its Complex Relationship With Rheumatoid Arthritis

Several unmet needs in PR may be identified: (1) concerns about the clinical case definition and diagnostic criteria, (2) the enigmatic and not well-understood relationship with RA, and (3) the best management and therapeutic strategy to improve acute attacks and prevent the evolution to RA.

### Diagnosis and Case Definition

The case definition of PR included in various studies vary due to the differing inclusion criteria used because of the lack of validated clinical criteria. In most studies, the criteria were *ad hoc*. All patients with multiple episodes of relapsing/remitting arthritis/periarthritis should be considered as PR patients, since no alternative diagnosis is found. However, there is no clear answer to this question. In our opinion, the clinical phenotype and other findings, such autoantibody positivity/negativity should alert clinicians to diagnoses other than PR, with a different disease evolution (10) (Table 4). Therefore, there is a need for consensual classification criteria for PR, which is not an easy task because, as stated, PR is probably more a syndrome than a disease. However, classification or diagnostic criteria may be useful in ascertaining the prognosis, evaluating the exact risk for progression to RA (or other rheumatic conditions), carrying out well-designed clinical trials and improving the disease management.

**Table 4:** Clinical and laboratory data in patients with recurring/relapsing arthritis that should alert the clinician to possible diagnoses other than palindromic rheumatism [modified from Cabrera-Villalba and Sanmarti R (10)].

Most acute episodes lasting more than 72 h, especially if more than one per week
Fever or prominent general symptoms
Oligoarticular/polyarticular attacks
Elevated acute phase reactants: ESR or CRP, especially during the intercrisis
The absence of autoantibodies (Rheumatoid factor or ACPA)
Accompanying symptomatology (skin lesions, intestinal symptoms, serositis, etc.).

### Is PR Simply a Pre-RA Stage?

We know that most patients with a presentation of PR, especially those with autoantibodies, evolve to RA. This suggests that, in most patients, PR is simply a pre-RA stage. In our opinion, PR emerges, even given the aforementioned problems with the case definition, as a definite clinical entity that is different and probably more homogeneous than the so-called inflammatory arthralgia or suspected arthralgia for progression to RA (41). But what accounts for patients who do not evolve to RA in the long term and persist as PR or are in partial or total remission, including patients with high ACPA titers? Why do these patients have this curious intermittent clinical phenotype? Is autoantibody-negative PR a separate entity, including genetic or pathogenetic pathways with a greater role played by autoinflammation rather than autoimmunity? Can the factors associated with progression to RA be correctly identified? These questions remain unanswered. Current data suggest most cases of PR may be considered part of the same continuum as RA, especially those with autoantibodies, although some will probably never evolve to persistent, chronic arthritis or will not fulfill current RA classification criteria. As commented, some recent studies have shown a more restricted immune response profile of ACPA and antiCarP in longstanding PR patients compared with established RA, as occurs in the preclinical phase of RA. The exact clinical significance of this finding is unknown but may explain why some patients with PR are less prone to progress to RA. Studies of the immune profile in recent-onset PR are warranted.

It is of great interest to determine the exact risk for progression to RA in a patient with typical recent-onset PR, such as our clinical case. Searching for these factors in large homogeneous populations with a long observation period, and including genetic studies, would be of interest. Imaging studies confirming the differing pattern of inflammation compared with RA during acute attacks in recent-onset PR are of interest and their prognostic significance in the long-term follow up merits further investigation.

### Management

As stated, the management and treatment of PR is completely empirical at present, and only antimalarials have been shown to reduce the frequency and intensity of acute attacks. PR may present a unique opportunity for therapeutic interventions that avoid the frequent progression to chronic polyarthritis. Randomized clinical trials with potentially-effective drugs in PR patients with a high risk for RA progression are warranted. However, recruitment difficulties may be of concern, taking into account the intermittent nature of PR and its relatively-low prevalence. The most rational drug to use in these patients and in clinical trials is difficult to establish on the basis of current knowledge of the mechanisms involved in PR and the exact risk for progression to RA. Biological therapies which modulate the B or T cell response, such as rituximab or abatacept, may avoid progression to persistent arthritis, as already tested in patients with pre-RA or inflammatory arthralgia (42). Since an autoinflammatory component may operate in PR, Il-1 blockade may be rational, at least in possibly seronegative patients.

In reference to our clinical case, the exact probability of evolving to RA is unknown although it is high because the patient has several predictive factors for evolution to RA. We think that, currently, a therapeutic strategy is mandatory and, on the basis of recent knowledge, antimalarials seem to be the most appropriate therapy for this patient. High titers of positive ACPAs does not necessarily imply that the patient will develop or can be classified as RA, so in our opinion, treatment with methotrexate or other csDMARDs is questionable. The above evidence clearly shows there is still a great deal to cover in the management and treatment of PR.

## Data Availability Statement

The original contributions presented in the study are included in the article/supplementary material, further inquiries can be directed to the corresponding author/s.

## Author Contributions

RS, JC, and IH contributed to the conception and manuscript design and wrote the first version of the manuscript. RC-M, SC-V, JR, GS, BF-S, and RM takes part of the different studies referenced in the manuscript and revised it critically. All authors contributed to the article and approved the submitted version.

## Conflict of Interest

The authors declare that the research was conducted in the absence of any commercial or financial relationships that could be construed as a potential conflict of interest.
